# Harnessing Hazara Virus as a Surrogate for Crimean–Congo Hemorrhagic Fever Virus Enables Inactivation Studies at a Low Biosafety Level

**DOI:** 10.3390/pathogens14070700

**Published:** 2025-07-15

**Authors:** Judith Olejnik, Kristina Meier, Jarod N. Herrera, Daniel J. DeStasio, Dylan J. Deeney, Elizabeth Y. Flores, Mitchell R. White, Adam J. Hume, Elke Mühlberger

**Affiliations:** 1 Department of Virology, Immunology and Microbiology, Chobanian & Avedisian School of Medicine, Boston University, Boston, MA 02118, USA; jolejnik@bu.edu (J.O.); krmeier@bu.edu (K.M.); jherrera@bu.edu (J.N.H.); ddest@bu.edu (D.J.D.); ddeeney@bu.edu (D.J.D.); mitchw@bu.edu (M.R.W.); 2 National Emerging Infectious Diseases Laboratories (NEIDL), Boston University, Boston, MA 02218, USA; eyflores@bu.edu; 3 Center for Regenerative Medicine (CReM), Boston University and Boston Medical Center, Boston, MA 02118, USA

**Keywords:** virus inactivation, TRIzol, aldehydes, Hazara virus, Crimean-Congo hemorrhagic fever virus, negative sense RNA viruses, biosafety level 4

## Abstract

Research on highly pathogenic biosafety level 4 (BSL-4) viruses that are classified as Select Agents involves transferring inactivated materials to lower containment levels for further analysis. Compliance with Select Agent and BSL-4 safety regulations necessitates the validation and verification of inactivation procedures. To streamline this process, it would be beneficial to use surrogate BSL-2 viruses for inactivation studies. This not only simplifies BSL-4 work but also enables the testing and validation of inactivation procedures in research facilities that lack access to high-containment laboratories yet may receive samples containing highly pathogenic viruses that require efficient and complete inactivation. In this study, we used Hazara virus (HAZV) as a surrogate virus for Crimean–Congo hemorrhagic fever virus to show the efficacy of various inactivation methods. We demonstrate the successful inactivation of HAZV using TRIzol/TRIzol LS and aldehyde fixation. Importantly, the parameters of the aldehyde inactivation of cell pellets differed from those of the monolayers, highlighting the importance of inactivation validation. As part of this study, we also defined specific criteria that must be met by a BSL-2 virus to be used as a surrogate for a closely related BSL-4 virus. Defining these criteria helps identify suitable nonpathogenic surrogates for developing inactivation procedures for highly pathogenic viruses.

## 1. Introduction

Crimean–Congo hemorrhagic fever virus (CCHFV; *Orthonairovirus haemorrhagiae*) causes severe disease in humans, with case fatality rates up to 40% [1,2]. CCHFV is an arbovirus that is transmitted through ticks of the *Ixodidae* family. Changes in the habitat of the tick vectors have led to the emergence of CCHFV in previously unaffected areas. Cases of Crimean–Congo hemorrhagic fever (CCHF) have been reported in multiple countries across Africa, the Balkans, the Middle East, Asia, and southern Europe [3,4,5,6]. Due to the high case fatality rates, the lack of countermeasures and vaccines, and potential human-to-human transmissibility, CCHFV is classified as a risk group 3 or 4 pathogen in most countries and must be handled at high or maximum containment [7]. For example, in the United States, work with CCHV must be performed at biosafety level 4 (BSL-4). CCHFV is also classified as a Select Agent by the United Sates Federal Select Agent Program (FSAP), which is overseen by the Centers for Disease Control and Prevention/Division of Select Agents and Toxins and the Animal and Plant Health Inspection Service/Division of Agricultural Select Agents and Toxins. Similarly, work with CCHFV in some countries, including Canada, United Kingdom, France, Sweden, Germany, Switzerland, Italy, Kazakhstan, Georgia, South Africa, and Australia, must be performed at BSL-4, containment level 4 (CL4), or physical containment level 4 (PC4), while work with CCHFV in other, mostly endemic countries, including Turkey, Slovenia, Albania, Bulgaria, Greece, and Senegal, is classified as BSL-3 [7]. Few countries permit work with CCHFV at BSL-2 [7]. Additional regulations akin to Select Agent regulations regarding the use of CCHFV are also in place in other countries, including in the United Kingdom, as outlined in Schedule 5 of the Anti-terrorism, Crime, and Security Act 2001, and in France, as outlined in regulations for transport, handling, and storage of microorganisms and toxins (Microorganismes et toxines hautement pathogènes—MOT), which are enforced by the French National Agency for Medicines and Health Products Safety (ANSM).

CCHFV belongs to the *Nairoviridae* family within the order *Hareavirales* of the *Bunyaviricetes* class [2,8]. Common features of nairoviruses include a lipid envelope and a segmented negative-sense RNA genome that is divided into three segments. Hazara virus (HAZV), another member of the *Nairoviridae* family, was first isolated from *Ixodes redikorzevi* ticks in Pakistan and is closely related to CCHFV [9]. Both viruses belong to the genus *Orthonairovirus* and show a high degree of similarity regarding their genome organization, virion structure, and the structure and function of the viral proteins [10,11,12,13,14,15,16]. However, in contrast to CCHFV, there are no reported symptomatic human cases of HAZV infection. It was therefore concluded that HAZV does not pose a risk to human health. For this reason, HAZV is classified as a risk group 2 agent and can be handled under standard BSL-2 conditions [17]. Due to its close relationship to CCHFV, HAZV has been widely used as a surrogate virus for CCHFV [12,18,19,20,21,22,23,24,25,26,27,28], including for inactivation studies [29].

FSAP has implemented strict regulations to ensure that material containing Select Agents is rendered non-infectious beyond detection limits before the material can be removed from high or maximum containment. These regulations include in-house (at each facility) validation or verification of the applied inactivation procedures [30]. Importantly, FSAP regulations permit the use of viruses from the same family as suitable surrogates for Select Agent viruses, such as Ebola virus (EBOV) as a representative for all filoviruses [30]. While specific language varies from country to country, regulations regarding the inactivation of BSL-4 (or CL4/PC4) agents remain similar. For example, the Canadian Biosafety Standard section 4.5.18 outlines that inactivation procedures must be “validated and routinely verified” and numerous guidelines for European maximum containment laboratories require similarly rigorous inactivation testing, including limit of detection analyses, use of control samples, and challenge samples when using cytotoxic chemicals for inactivation [31,32].

In our previous work describing various inactivation procedures for BSL-4 viruses, we used EBOV as our representative for the filovirus family, Nipah virus (NiV) for the paramyxovirus family, and Lassa virus (LASV) for the arenavirus family [33,34,35]. Since these surrogates are classified as BSL-4 viruses, the inactivation studies had to be performed within BSL-4 containment. Under these conditions, even procedures that can be easily carried out with inactivated material, such as immunofluorescence analysis (IFA), must be carried out in maximum containment until the respective inactivation method has been approved. To minimize the time in the BSL-4 laboratory and streamline the validation of inactivation procedures, we chose HAZV, a BSL-2 virus, as our surrogate virus for the nairovirus family. To cover a wide range of sample types, it is desirable to generate samples with high viral loads to validate inactivation procedures. In addition, reliable detection methods to visualize viral infection are required to show efficient inactivation [35]. In our previous work, we relied on the virus-induced cytopathic effect (CPE) and the use of recombinant viruses expressing fluorescent proteins as detection methods, as well as IFA using virus-specific antibodies for the final set of samples [33,34,35]. Some studies utilize RT-qPCR as a measure of viral kinetics during virus inactivation testing. However, RT-qPCR cannot be used to discern between RNA from replicating versus non-replicating (i.e., inactivated) virus. Therefore, although decreasing amounts of viral RNA from one passage to the next is indicative of some level of viral inactivation, it does not exclude the possibility of small amounts of replicating virus. Along similar lines, the detection of viral RNA by RT-qPCR is not necessarily indicative of replicating virus. A recent study on NiV inactivation showed that some of the inactivated samples that did not contain any infectious virus still resulted in weak positive results in the RT-qPCR analysis [36]. Due to this inability of RT-qPCR to discriminate between infectious and non-infectious virus, we refrained from using this method throughout our inactivation studies.

HAZV reaches high viral titers in cell culture systems [27,37], which allows for high viral loads in the inactivation studies. In addition, recombinant HAZV expressing GFP was made available to us, facilitating the reliable detection of the infected cells [22]. In this study, we used HAZV-GFP to verify and validate the inactivation of nairovirus-infected cells by TRIzol, TRIzol LS, 4% paraformaldehyde, and 10% formalin.

## 2. Materials and Methods

### 2.1. Cell Lines

African green monkey kidney cells (Vero E6; ATCC, Manassas, VA, USA; CRL-1586) were maintained in Dulbecco’s modified Eagle medium (DMEM; Gibco/Thermo Fisher Scientific, Waltham, MA, USA) supplemented with L-glutamine (200 mM; Thermo Fisher Scientific, Waltham, MA, USA) and 10% fetal bovine serum (FBS; R&D Systems, Minneapolis, MN, USA). Cell culture medium was supplemented with 100 µg/mL Primocin (Invivogen, San Diego, CA, USA). Cells were grown at 37 °C and 5% CO_2_.

### 2.2. Virus Propagation

HAZV-expressing GFP (HAZV-GFP) was kindly provided by J. N. Barr, University of Leeds, UK [22]. The eGFP gene was inserted into the S segment separated from the nucleoprotein (N) gene by a porcine teschovirus-1 2A peptide linker (P2A) [22]. HAZV-GFP was propagated in Vero E6 cells in cell culture medium supplemented with 2% FBS. Virus titers were determined in Vero E6 cells by the tissue culture infectious dose 50 (TCID_50_) assay using the Spearman and Kärber algorithm [38,39]. The titer of the HAZV stock used for the inactivation studies was 9.1 × 10^7^ TCID_50_/mL.

### 2.3. Inactivation of HAZV-GFP-Infected Cells Using TRIzol

A total of 9.4 × 10^6^ Vero E6 cells seeded in T175 flasks were mock-infected or infected with HAZV-GFP at a multiplicity of infection (MOI) of 0.05. At 3 days post-infection (dpi), when complete infection was observed, cell supernatants were removed, and the cells were scraped into 20 mL of phosphate buffered saline (PBS; Thermo Fisher Scientific, Waltham, MA, USA), transferred into tubes, pelleted by low-speed centrifugation, and resuspended in 1 mL DMEM or TRIzol (Thermo Fisher Scientific, Waltham, MA, USA). To determine the cell number in the T175 flasks at the time of inactivation, Vero E6 cells were seeded in an extra T175 flask, incubated for the same time, and counted. The DMEM and TRIzol samples were vortexed, incubated for 10 min at room temperature, and purified using size exclusion columns (Amicon Ultra-0.5 Centrifugal Filter Unit 10 kDa; Millipore-Sigma, Burlington, MA, USA) as previously described [34,35]. The samples were eluted in 0.5 mL of PBS, and each eluate was used for the infection of 1.8 × 10^7^ Vero E6 cells seeded in T175 flasks. For the challenge samples, HAZV-GFP was mixed with the eluate obtained from TRIzol-treated non-infected cells, and this mixture was used to infect 2 × 10^7^ Vero E6 cells seeded in T175 flasks at an MOI of 0.05. After an incubation time of 4 days at 37 °C and 5% CO_2_, the entire cell supernatants were clarified by low-speed centrifugation and used to infect Vero E6 cells seeded in T175 flasks. At 3 dpi, cell supernatants were clarified by low-speed centrifugation, and 0.2 mL was used to infect Vero E6 cells seeded in a 96-well plate. At 2 dpi, the cells were fixed with 10% formalin (LabChem, Zelienople, PA, USA). IFA using virus-specific antibodies was performed to determine infection rates.

### 2.4. Inactivation of rHAZV-GFP Particles Using TRIzol LS

A total of 250 µL of HAZV-GFP stock solution (2.275 × 10^7^ TCID_50_ units) in DMEM supplemented with 2% FBS or 250 µL of DMEM supplemented with 2% FBS were mixed with 750 µL of TRIzol LS (Thermo Fisher Scientific, Waltham, MA, USA) in a 1:3 (*v*/*v*) ratio, as recommended by the manufacturer. The samples were vortexed, incubated for 10 min at room temperature, purified using size exclusion columns (Amicon Ultra-0.5 Centrifugal Filter Unit 10 kDa) [34,35], and eluted in 0.5 mL of PBS. The entire eluates were used to infect 2 × 10^7^ Vero E6 cells seeded in T175 flasks. For the challenge samples, HAZV-GFP was mixed with column-purified eluate from the TRIzol LS-treated cell culture medium (DMEM + 2% FBS), and the mixture was used to infect Vero E6 cells seeded in T175 flasks at an MOI of 0.05. Passaging to a second T175 flask, the monitoring of cells, infection of a 96-well plate, and IFA were performed as described for TRIzol testing.

### 2.5. Inactivation of HAZV-GFP-Infected Cell Monolayers Using Aldehydes

A total of 9.4 × 10^6^ Vero E6 cells seeded in T175 flasks were mock-infected or infected with HAZV-GFP at an MOI of 0.05. At 3 dpi, when the complete infection of cells was observed, cell supernatants were removed and replaced with 35 mL (200 µL per cm^2^ of cell monolayer) of 10% buffered formalin (LabChem, Zelienople, PA, USA), 4% paraformaldehyde (PFA; Thermo Fisher Scientific, Waltham, MA, USA), or PBS. Flasks were rotated to ensure that the fixatives coated all interior surfaces and incubated for 30 min or 6 h at 4 °C. After incubation, the fixative or PBS was removed, and cells were rigorously washed four times with 20 mL of PBS to eliminate the cytotoxic effects of the used fixatives. Cells were scraped into 20 mL of PBS, transferred into tubes, pelleted by low-speed centrifugation, resuspended in 1 mL PBS, and used to infect Vero E6 cells seeded in T175 flasks the day prior.

To determine the cell numbers at the time of inactivation, 9.4 × 10^6^ Vero E6 were seeded in an extra T175 flask at the time of infection, incubated for 3 days, and counted. For the challenge samples, HAZV-GFP was mixed with formalin- or PFA-treated non-infected cells and used to infect cells at an MOI of 0.05. The challenge samples were included to show that HAZV-GFP can replicate in cells that were exposed to formalin- or PFA-treated cells. Passaging to a second T175 flask, the infection of a 96-well plate and virus-specific IFA were performed as described for TRIzol testing.

### 2.6. Inactivation of HAZV-Infected Cell Pellets Using Aldehydes

Vero E6 cells seeded in T175 (9.4 × 10^6^ cells) or T75 flasks (4 × 10^6^ cells) were mock-infected or infected with HAZV-GFP at an MOI of 0.05 and processed at specific time points, as described in the paragraph above. Cells were scraped and pelleted for 10 min at 500× *g* and 4 °C. After removing the cell culture medium, the cell pellets were overlaid with 2 mL of 10% formalin, 4% PFA, or PBS. The cell pellets remained intact and were not mixed or vortexed. After an incubation time of 30 min, 1 h, 3 h, or 6 h at 4 °C, the cell pellets were washed 4 times with 2 mL PBS, resuspended in 1 mL DMEM, and used to infect Vero E6 cells seeded in T75 flasks, as described in the paragraph above, including an HAZV-GFP challenge sample. Passaging to a second T175 or T75 flask (as used for initial infection), the infection of a 96-well plate and IFA were performed as described for TRIzol testing.

### 2.7. Limit of Detection Analysis

A total of 1, 10, or 100 TCID_50_ units of HAZV-GFP diluted in a cell culture medium supplemented with 2% FBS were added to a T75 or T175 flask of Vero E6 cells. At 4 dpi, the entirety of supernatants from the initial flasks was transferred to a second T175 or T75 flask of Vero E6 cells. The cells were assessed for CPE and/or fluorescence. At 3 dpi, 200 µL of each supernatant was used to infected Vero E6 cells seeded in 96-well plates. At 2 dpi, the cells were fixed with 4% PFA and used for IFA.

### 2.8. Immunofluorescence Analysis

A total of 2 × 10^4^ Vero E6 cells seeded per well of a 96-well plate were infected with 200 µL of cell supernatant (3 wells per test sample). The infected cells were incubated for 2 days at 37 °C and 5% CO_2_, and fixed with 4% PFA. To determine the initial infection rates, 1 × 10^5^ Vero E6 cells seeded in 8-well ibidi µ-slides (Thermo Fisher Scientific, Waltham, MA, USA) were infected at an MOI of 0.05 with HAZV-GFP and fixed with 4% PFA after 4 days. The fixed cells were permeabilized with 0.1% Triton X100 (Boston Bioproducts, Milford, MA, USA) for 10 min at room temperature, incubated in 0.1 M glycine (Boston Bioproducts, Milford, MA, USA) for 5 min at room temperature, and subsequently incubated in blocking buffer (2% bovine serum albumin, 0.2% Tween 20, 3% glycerol, and 0.05% sodium azide in PBS) for 20–60 min at room temperature. After each step, the cells were washed three times in PBS. Cells were incubated overnight at 4 °C with a goat polyclonal antiserum directed against the HAZV nucleoprotein (N) (kindly provided by John N. Barr, University of Leeds, Leeds, UK). The cells were washed four times in PBS and incubated with an Alexa Fluor 594 conjugated anti-sheep secondary antibody plus 4′,6-diamidino-2-phenylindole (DAPI; Sigma-Aldrich at 200 ng/mL for nuclei staining) for 1 h at room temperature. Images were acquired using a Nikon TS100 Eclipse microscope and Nikon DS Qi1Mc camera with the NIS Elements F software (version 4.60.00) or with a Nikon Eclipse Ti2 microscope with a Photometrics Prime BSI camera and the NIS Elements AR software (version 5.42.05). The Fiji Image J software (downloaded from https://github.com/fiji) was used to create merge channel images and add scale bars.

## 3. Results

### 3.1. Reliable and Sensitive Detection of HAZV-GFP Infection

#### 3.1.1. Detection of HAZV-GFP Infection

**Figure 1:**
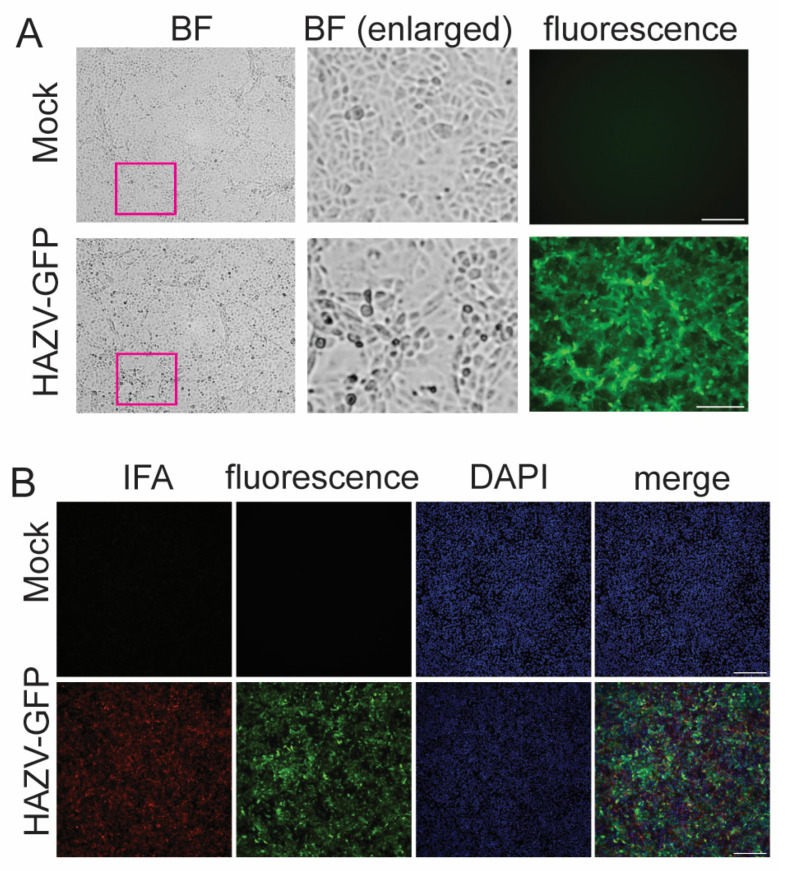
Detection of HAZV-GFP infection. A total of 1 × 10^5^ Vero E6 cells seeded in chamber slides were infected with HAZV-GFP at an MOI of 0.05 and incubated for 4 days. (**A**) Live-cell imaging of infected cells. BF, brightfield. Middle panels show enlarged areas of pink insets. (**B**) Immunofluorescence analysis of HAZV-GFP-infected Vero E6 cells. Cells were fixed and stained with an antibody detecting the HAZV N protein (red). GFP expression is shown in green. Cell nuclei are stained with DAPI (blue). Scale bar, 250 µm. The experiment was performed at least 6 times.

Validating viral inactivation methods requires the reliable and sensitive detection of viral infection. HAZV-GFP contains an additional transcription unit encoding GFP, which allows to visualize infected cells through green fluorescence [22]. In addition to GFP fluorescence, the induction of CPE and IFA using an anti-HAZV N antibody were used to determine HAZV-GFP infection. Cells infected with HAZV-GFP were easily identified by green fluorescence due to GFP expression even in the absence of CPE (Figure 1A). Compared to our previous inactivation studies with EBOV, NiV, and SARS-CoV-2 [34,35], CPE induced by HAZV infection was generally milder (Figure 1A, enlarged images). Due to the lack of pronounced CPE in HAZV-GFP-infected Vero E6 cells, we did not rely on CPE alone as a readout for determining infection in our inactivation studies.

In addition, infection was confirmed by IFA, as shown by the colocalization of red and green cells (Figure 1B). For the inactivation studies, IFA staining was only performed on samples used to determine the initial infection rate and the final infections in the 96-well plates because immunostaining to visualize HAZV-GFP-infected cells required fixation, which precluded the further incubation and analysis of the samples.

#### 3.1.2. Results of Limit of Detection Analysis

**Figure 2:**
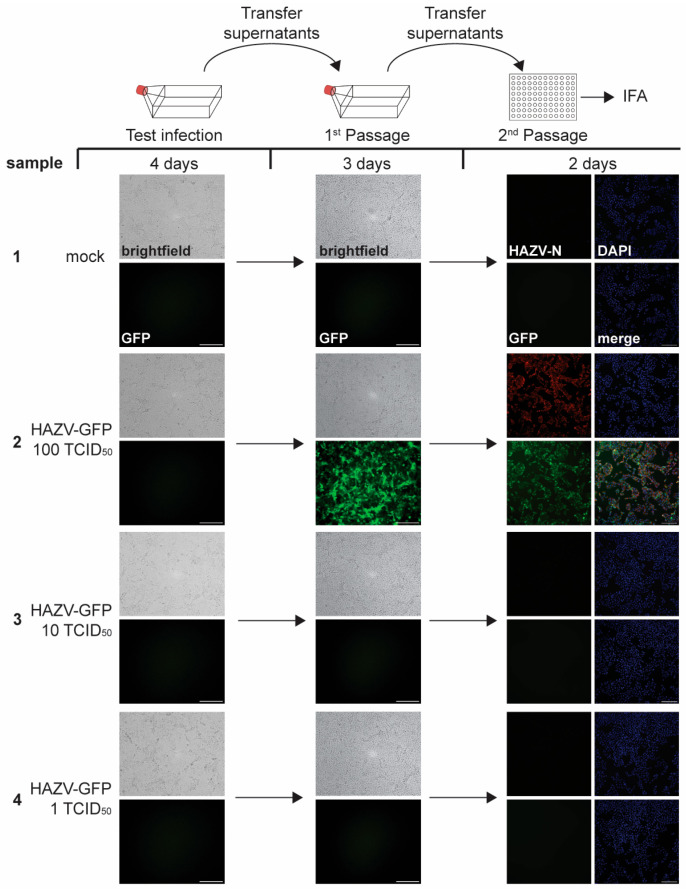
Limit of detection analysis for HAZV-GFP. Top, schematic of the assay. Vero E6 cells seeded in T175 flasks were mock-infected or infected with 1, 10, or 100 TCID_50_ units of HAZV-GFP. Fluorescence and brightfield images were taken at 4 dpi to monitor GFP fluorescence and CPE (Test infection). Clarified supernatants were passaged onto Vero E6 cells seeded in T175 flasks. Cells were incubated for 3 days and monitored for viral infection (1st Passage). Clarified supernatants were then used to infect Vero E6 cells seeded in 96-well plates and fixed at 2 dpi. IFA was performed using an anti-HAZV N antibody (red, 2nd Passage). Cell nuclei were stained with DAPI (blue). Scale bars, 250 µm. Image labeling for all samples as shown for sample 1. The experiment was performed six times. The results are summarized in Table 1.

After establishing reliable viral detection assays, we next tested if our inactivation validation protocols were sufficiently sensitive to detect small amounts of infectious HAZV-GFP. For this, we performed limit of detection (LOD) analyses alongside our inactivation testing. Vero E6 cells seeded in T175 or T75 flasks were infected with high dilutions of HAZV-GFP stock aiming for an infection dose of 1, 10, or 100 infectious TCID_50_ units (Figure 2). The infected cells were monitored for signs of infection for 4 days; then, supernatants were clarified and passaged onto fresh cells seeded in flasks. At 3 dpi, a second passage was performed onto cells seeded in 96-well plates. Plates were fixed at 2 dpi and analyzed by IFA for the presence of HAZV N in addition to GFP fluorescence to detect HAZV-GFP infection. We performed the LOD analysis in parallel to all tested inactivation procedures, and the results of one study are shown in Figure 2. Due to the low amount of virus, we did not observe CPE or GFP fluorescence in the test infection, while mild CPE and strong GFP fluorescence were detected in the first passage for the 100 TCID_50_ unit sample with the subsequent positive HAZV-N staining by immunofluorescence in the second passage (Figure 2). We reliably observed infection when we used 100 TCID_50_ units of HAZV-GFP as the inoculum in all performed assays. In two out of six infections, we detected a HAZV-GFP infection when the cells were infected with 1 TCID_50_ unit (Table 1). These analyses ensure that a lack of virus detection in the inactivation validation studies is due to the successful inactivation of HAZV-GFP below the LOD limit.

### 3.2. TRIzol and TRIzol LS Inactivation of HAZV-GFP

TRIzol and TRIzol LS are widely used for the inactivation of virus-containing cells or solutions for the purpose of RNA extraction. TRIzol and TRIzol LS are monophasic mixtures of phenol CAS # 108-95-2, guanidine isothiocyanate CAS # 593-84-0, and ammonium thiocyanate CAS # 1762-95-4 in slightly different ratios and are highly virucidal.

#### 3.2.1. TRIzol Inactivation

**Figure 3:**
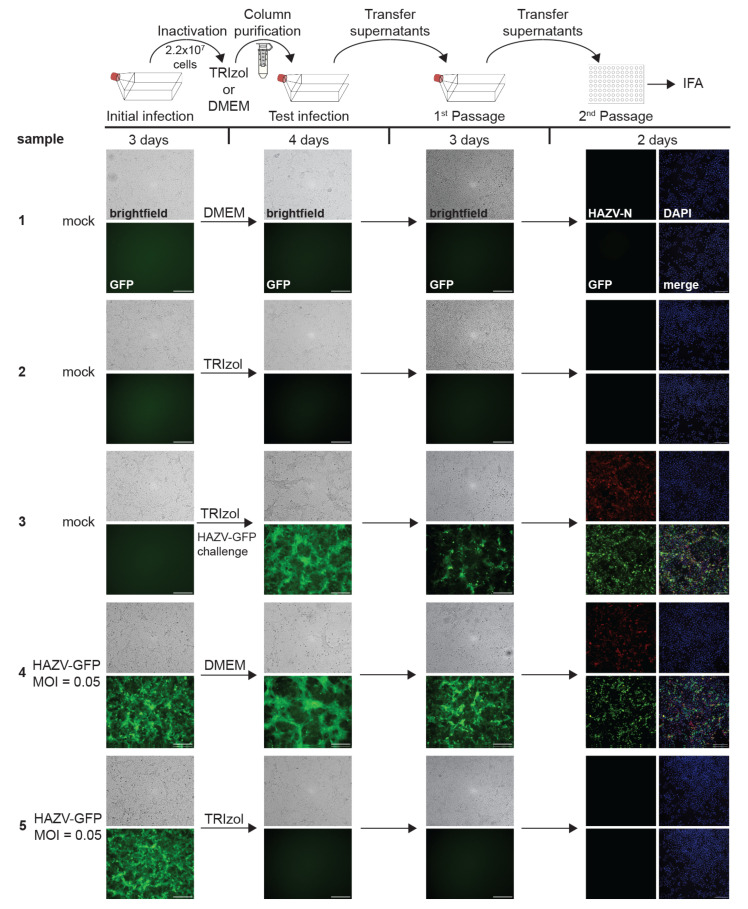
Inactivation of HAZV-GFP with TRIzol. Top, schematic of the assay. Vero E6 cells seeded in T175 flasks were mock-infected or infected with HAZV-GFP at an MOI of 0.05. At 3 dpi, fluorescence and brightfield images were taken to assess the presence of GFP and CPE in samples as a marker for viral infection (initial infection). Cells were harvested in either TRIzol or DMEM, column-purified, and transferred onto Vero E6 cells seeded in T175 flasks. Challenge samples were infected with HAZV-GFP at MOI 0.05. Samples were monitored for GFP fluorescence and CPE for 4 days (test infection). Clarified supernatants were passaged onto Vero E6 cells seeded in T175 flasks. Cells were incubated for 3 days and monitored for viral infection (1st Passage). Clarified supernatants were then used to infect Vero E6 cells seeded in 96-well plates and fixed at 2 dpi. IFA was performed using an anti-HAZV N antibody (red, 2nd Passage). Cell nuclei were stained with DAPI (blue). Scale bars, 250 µm. Image labeling for all samples as shown for sample 1. The experiment was performed twice with similar outcomes.

To validate the inactivation of HAZV-GFP in infected cells by TRIzol, we adapted our established testing procedure [35] for optimal time points to use with HAZV-GFP. Briefly, T175 flasks infected with HAZV-GFP at an MOI of 0.05 were processed at 3 dpi, when complete infection was observed by GFP fluorescence (Figure 3, initial infection). The cell number at the time of inactivation was 2.2 × 10^7^ cells per flask as determined by counting the cells from an extra T175 flask. In addition, we confirmed HAZV-GFP infection by IFA using chamber slides seeded with Vero E6 cells that were infected with HAZV-GFP at the same MOI in parallel to the flasks. The IFA results confirm complete infection (Figure S1). The cells in the flasks were scraped, pelleted by centrifugation, and the cell pellets resuspended in 1 mL of TRIzol or DMEM (negative control). After a 10-min incubation at room temperature, the samples were column-purified to eliminate toxicity. It has been demonstrated in numerous inactivation studies and across different virus families that Amicon columns are suitable to remove TRIzol and TRIzol LS from samples without significant virus loss [34,35,36,40,41,42]. Vero E6 cells seeded in T175 flasks were infected with eluates from the columns and monitored for viral infection (Figure 3, test infection). At 4 dpi, the entirety of the supernatants was then transferred onto fresh cells seeded in T175 cells (Figure 3, first passage) and then passaged a second time onto cells seeded in 96-well plates (Figure 3, second passage). All flasks and plates were monitored for signs of infection. The cells seeded in the 96-well plates were additionally fixed and stained for HAZV N by IFA.

As expected, the mock sample (Figure 3, sample 1) showed no signs of infection, whereas there was robust HAZV-GFP infection in the positive control, as demonstrated by GFP fluorescence, mild CPE, and HAZV-specific antibody staining (Figure 3, sample 4). The column purification successfully removed toxicity, as demonstrated by the lack of cell death in the TRIzol-treated mock sample (Figure 3, sample 2). Ample HAZV-GFP infection was observed in the challenge sample (Figure 3, sample 3), indicating that the exposure of cells to TRIzol-treated, column-purified eluates did not inhibit the ability of these cells to be subsequently infected. No GFP fluorescence, CPE, or HAZV-N-positive cells were observed in the samples treated with column eluates from TRIzol-treated, HAZV-GFP-infected cells, demonstrating the lack of infectious virus (Figure 3, sample 5). We developed an inactivation SOP based on these data that defines the inactivation condition for nairovirus-infected cells. According to this SOP, a maximum of 2.2 × 10^7^ cells infected with nairoviruses can be inactivated with a minimum volume of 1 mL of TRIzol per 10 cm^2^ cell culture area, followed by vortexing and a 10-min incubation at room temperature.

#### 3.2.2. TRIzol LS Inactivation

**Figure 4:**
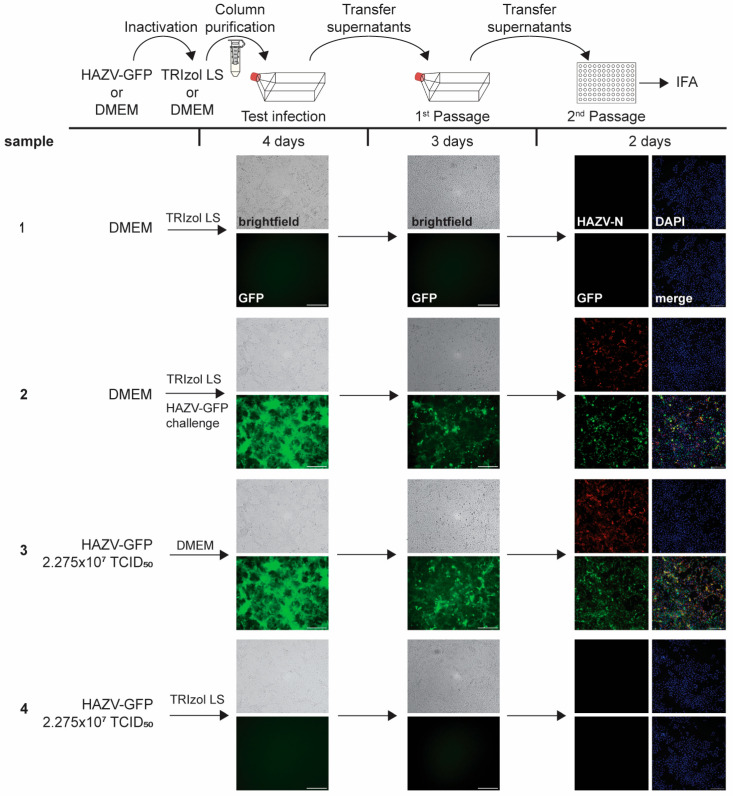
Inactivation of HAZV-GFP with TRIzol-LS. Top, schematic of the assay. Virus stock or DMEM were incubated with TRIzol-LS or DMEM, column-purified, and transferred onto Vero E6 cells seeded in T175 flasks. Challenge samples were infected with HAZV-GFP at MOI 0.05 (test infection). At 4 dpi, clarified supernatants were passaged onto Vero E6 cells seeded in T175 flasks. Cells were incubated for an additional 4 days and monitored for viral infection (1st Passage). Clarified supernatants were then used to infect Vero E6 cells seeded in 96-well plates. Cells were fixed at 2 dpi and stained with an anti-HAZV N antibody (red, 2nd Passage). Cell nuclei were stained with DAPI (blue). Scale bars, 250 µm. Image labeling for all samples as shown for sample 1. The experiment was performed twice with similar outcomes.

To establish an inactivation protocol for RNA extraction from cell supernatants containing the infectious virus, we tested the ability of TRIzol LS to inactivate HAZV-GFP particles. A high-titer virus stock solution was incubated with TRIzol LS, column-purified, and used to infect cells seeded in T175 flasks (Figure 4). A subsequent analysis was performed as described above for the TRIzol samples (Figure 3). While the mock samples did not show any signs of infection (Figure 4, sample 1), a robust HAZV-GFP infection was observed in the positive control (Figure 4, sample 3). The high-titer HAZV-GFP stock solution (2.275 × 10^7^ TCID_50_ units in a volume of 250 µL) treated with TRIzol LS was completely inactivated, as demonstrated by lack of GFP fluorescence, CPE, and HAZV-N staining (Figure 4, sample 4). Based on these data, we developed an inactivation SOP that defines the inactivation condition for nairovirus-containing supernatants. According to this SOP, the amount of infectious nairovirus particles in the solution must not exceed 2.27 × 10^7^ TCID_50_ units in a minimal volume of 250 µL. Inactivation is achieved through the addition of TRIzol LS at a ratio of 3:1 (3 volumes TRIZol LS per 1 sample volume) followed by vortexing and a 10-min incubation at room temperature.

### 3.3. Aldehyde Inactivation of HAZV-GFP

Aldehydes are routinely used to preserve cells for histochemistry, IFA, electron microscopy, and other applications. They are also known for their virucidal activity. In our study, we tested the ability of 10% buffered formalin and 4% PFA to inactivate HAZV-GFP-infected cell monolayers and cell pellets.

#### 3.3.1. Aldehyde Inactivation of HAZV-GFP-Infected Cell Monolayers

**Figure 5:**
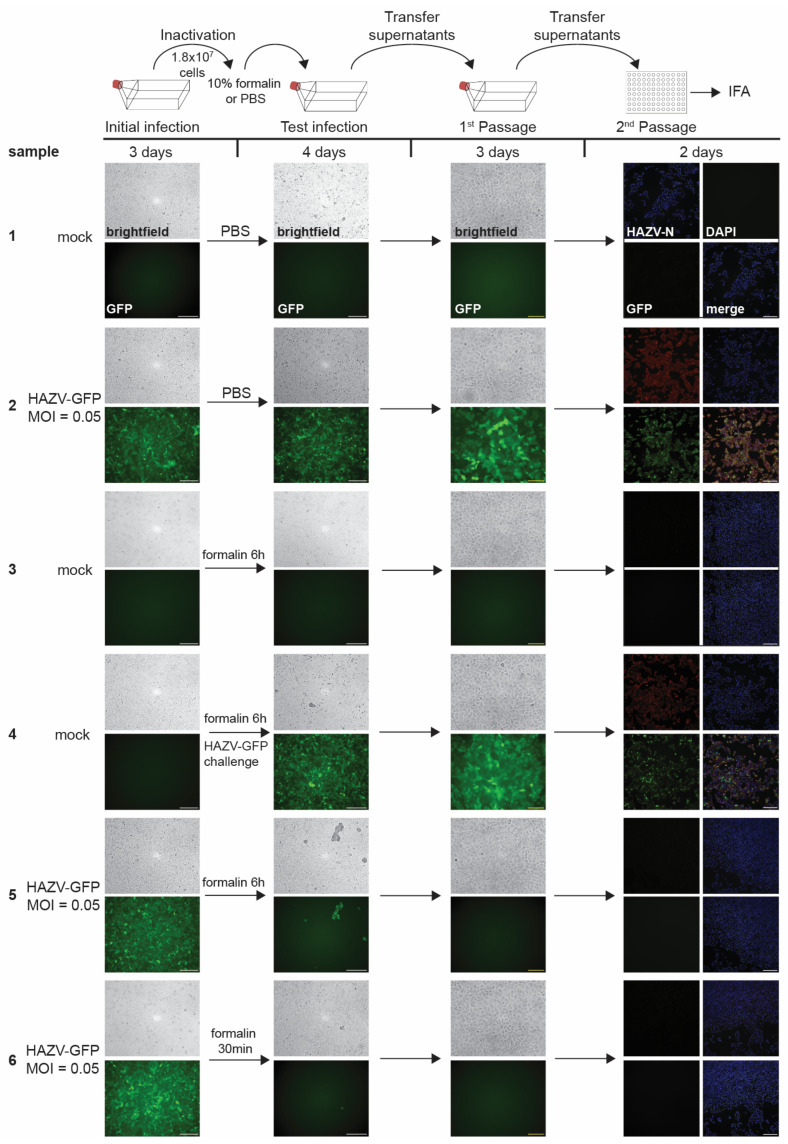
Inactivation of HAZV-GFP-infected cell monolayers with 10% formalin. Top, schematic of the assay. Vero E6 cells seeded in T175 flasks were mock-infected or infected with HAZV-GFP at an MOI of 0.05. At 3 dpi, fluorescence and brightfield images were taken to assess GFP fluorescence and CPE as markers for viral infection (Initial infection). Cells were incubated with 10% formalin or PBS (control). Treated cells were washed, pelleted, and transferred onto Vero E6 cells seeded in T175 flasks (test infection). Challenge samples were infected with HAZV-GFP at an MOI of 0.05. At 4 dpi, clarified supernatants were passaged onto Vero E6 cells seeded in T175 flasks. Cells were incubated for an additional 3 days and monitored for viral infection (1st passage). Note that images for the 1st passage were taken at a higher magnification. Clarified supernatants were then used to infect Vero E6 cells seeded in 96-well plates and fixed at 2 dpi. IFA was performed using an anti-HAZV N antibody (red, 2nd passage). Cell nuclei were stained with DAPI (blue). White scale bars, 250 µm; yellow scale bars in 1st passage column, 100 µm. Image labeling for all samples as shown for sample 1. The experiment was performed twice with similar outcomes.

To validate the inactivation of HAZV-GFP-infected cell monolayers with 10% formalin and 4% PFA, Vero E6 cells seeded in T175 flasks were infected with HAZV-GFP or left uninfected as controls. At 3 dpi, when complete infection was observed, the cells were fixed with 10% formalin or 4% PFA for 30 min or 6 h at 4 °C (Figure 5 and Figure S2, top schematic). PBS was used as a control that should not lead to virus inactivation. After the removal of the fixatives, the cells were washed multiple times, scraped, pelleted, resuspended in 1 mL PBS, and transferred onto fresh Vero E6 cells seeded in T175 flasks. Similar to the TRIzol inactivation study (Figure 3), multiple passages were performed to be able to detect low amounts of infectious virus. The results of formalin and PFA inactivation are shown in Figure 5 and Figure S2, respectively. In the positive control (Figure 5 and Figure S2, sample 2), mild CPE, GFP fluorescence, and positive HAZV-N IFA were observed throughout the experiment. Similarly, formalin- and PFA-fixed mock samples challenged with HAZV-GFP showed ample infection (Figure 5 and Figure S2, sample 4), demonstrating that the addition of PBS-washed aldehyde-fixed cells did not interfere with infection. After test infection with HAZV-GFP-infected cells treated with 10% formalin or 4% PFA, we observed GFP fluorescence, which colocalized with cell clumps observed on top of the monolayer in brightfield images (Figure 5 and Figure S2, samples 5 and 6, test infection). This is likely due to the preservation of GFP fluorescence in the infected cells after fixation, indicating that the GFP-positive cells represent fixed cells from the initial infection. Importantly, after the passaging of clarified supernatants, no GFP fluorescence was detected in any of the samples initially treated with HAZV-GFP-infected cells that had been fixed with 10% formalin or 4% PFA (Figure 5 and Figure S2, samples 5 and 6, first and second passages). This was confirmed by a lack of HAZV-N-positive staining using IFA (Figure 5 and Figure S2, second passage). This validates the ability of 10% formalin and 4% PFA to fully inactivate HAZV-GFP-infected monolayers after a 6-h (Figure 5 and Figure S2, sample 5) or 30-min (Figure 5 and Figure S2, sample 6) incubation at 4 °C. Based on this study, an SOP was developed that defines the inactivation conditions for nairoviruses. According to this SOP, a monolayer of maximal 1.8 × 10^7^ nairovirus-infected cells can be inactivated by incubation with 10% formalin or 4% PFA using at least 200 µL/cm^2^ surface area for at least 6 h at 4 °C before removal from high containment.

#### 3.3.2. Aldehyde Inactivation of HAZV-GFP-Infected Cell Pellets

**Figure 6:**
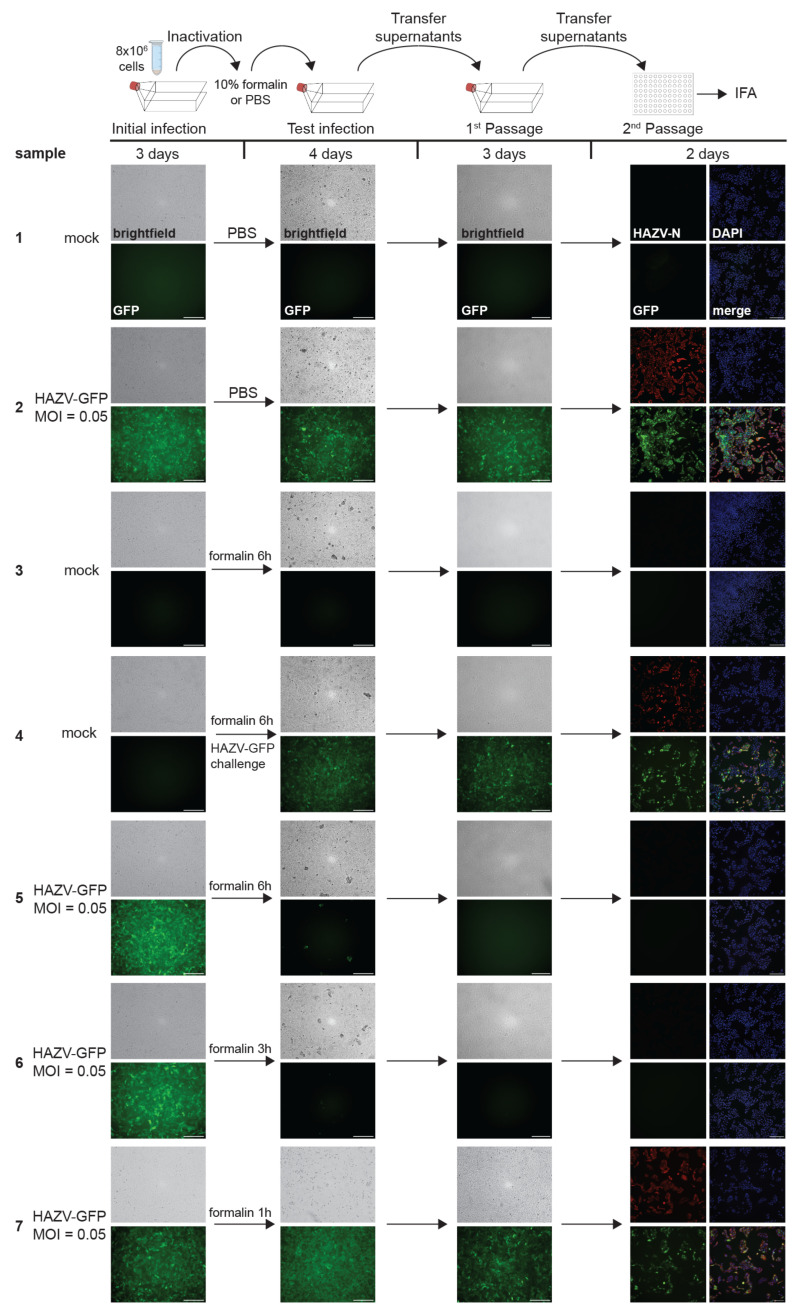
Inactivation of HAZV-GFP-infected cell pellets with 10% formalin. Top, schematic of the assay. Vero E6 cells seeded in T75 flasks were mock-infected or infected with HAZV-GFP at an MOI of 0.05. At 3 dpi, fluorescence and brightfield images were taken to assess the presence of GFP and CPE in samples as a marker for viral infection (initial infection). Cells were pelleted and overlaid with 10% formalin or PBS (control) for fixation. After the removal of the fixative, cell pellets were washed and transferred onto Vero E6 cells seeded in T75 flasks (test infection). Challenge samples were infected with HAZV-GFP at MOI 0.05. At 4 dpi, clarified supernatants were passaged onto Vero E6 cells seeded in T175 flasks (1st passage). At 3 dpi, clarified supernatants were then used to infect Vero E6 cells seeded in 96-well plates and fixed at 2 dpi. IFA was performed using an anti-HAZV N antibody (red, 2nd passage). Cell nuclei were stained with DAPI (blue). Scale bars, 250 µm. Image labeling for all samples as shown for sample 1. The experiment was performed twice with similar outcomes.

For some assays, such as the use of suspension cells, flow cytometry, or electron microscopy, the fixation of cell pellets instead of monolayers is required. Changes in the inactivation parameters require the separate testing of the used conditions to assure complete inactivation. To determine the parameters of the aldehyde inactivation of HAZV-GFP-infected cell pellets, we employed the same testing procedure as described above for monolayers, but the infected cells from a T75 flask were scraped and pelleted before fixation. Cell pellets were overlaid with 2 mL 10% formalin or 4% PFA. PBS was used as a control. Importantly, the cell pellets were not resuspended in the fixatives to provide the most stringent inactivation conditions. Resuspending the cell pellets would allow for a better penetration of the cells by the fixative. The cell pellets were incubated for the indicated times with fixative, rigorously washed, resuspended in PBS, and used to infect Vero E6 cells seeded in T75 cell (Figure 6 and Figure S3, schematics on top). We initially tested a high cell number (1.8 × 10^7^ cells at the time of inactivation) obtained from infected cells seeded in a T175 flask. However, in contrast to the results obtained with cell monolayers, HAZV-GFP was not inactivated by formalin or PFA treatment in the cell pellets after an incubation time of 30 min, 1 h, or 3 h. Since our inactivation procedures involve safety margins, complete inactivation must be shown by 3 h at the latest to develop an SOP for a minimal incubation time of 6 h. To avoid longer fixation times, which could lead to a loss of sensitivity for future analysis, we decreased the number of infected cells by using cell pellets obtained from T75 flasks (8 × 10^6^ cells at time of inactivation) for our studies. The results for the negative and positive controls as well as the viral challenge samples (Figure 6 and Figure S3; samples 1, 2, and 4) were in line with those observed for the monolayers (Figure 5 and Figure S2). The incubation of HAZV-GFP infected cell pellets with 10% formalin or 4% PFA for 3 or 6 h led to complete inactivation, as shown by the lack of GFP fluorescence, CPE, and HAZV-N staining (Figure 6 and Figure S3, samples 5 and 6). In contrast to the results obtained with the monolayers, a 1-h incubation with 10% formalin or 4% PFA failed to inactivate HAZV-GFP-infected cell pellets (Figure 6 and Figure S3, sample 7). This highlights the importance of testing the specific inactivation conditions, particularly regarding fixation time and cell numbers. Based on these results, an SOP was developed for inactivating cell pellets that restricts the maximum cell number to 8 × 10^6^ nairovirus-infected cells that must be incubated in a minimal volume of 2 mL of 10% formalin or 4% PFA for at least 6 h at 4 °C.

## 4. Discussion

This study aimed to validate inactivation methods for HAZV-GFP as a nonpathogenic surrogate for the nairovirus family, including CCHFV. We focused on basic inactivation methods for RNA purification and the fixation of cells for downstream analysis. Our data demonstrate the successful inactivation of HAZV-GFP particles by TRIzol LS and HAZV-GFP-infected cells by TRIzol after the recommended incubation time of 10 min at room temperature for downstream RNA analysis and are in line with previous work on inactivating BSL-4 viruses, including filoviruses, henipaviruses, and arenaviruses [35,36,43,44,45,46]. We are not aware of published inactivation studies explicitly describing the TRIzol inactivation of nairoviruses. However, TRIzol LS treatment was previously shown to inactivate Rift Valley fever virus, a member of the *Phenuiviridae* family within the order *Hareavirales* [42,45,46]. Of note, prior to the ICTV virus taxonomy revisions, CCHFV and Rift Valley fever virus were both members of the *Bunyaviridae* family. Due to the available inactivation data for bunyaviruses, TRIzol and TRIzol LS have routinely been used to inactivate CCHFV for viral RNA analysis [47,48,49,50].

Inactivation procedures were also validated for 10% formalin and 4% PFA fixation. While we observed the complete inactivation of 1.8 × 10^7^ HAZV-GFP-infected cell monolayers after an incubation time of 30 min at 4 °C with both 10% formalin and 4% PFA, this was different for cell pellets. When using high cell numbers (1.8 × 10^7^ cells) to generate infected cell pellets overlaid with the fixatives, we did not observe complete inactivation even after a 3-h incubation time. This is likely due to the slow penetration and fixation of the cell pellets, whereas the fixatives must only penetrate a single cell layer in the case of the cell monolayers. These data show that cell numbers, sample format (e.g., monolayer versus cell pellet), and aldehyde incubation time are critical parameters of complete fixative penetration and sample inactivation. Importantly, we did not observe significant differences in the ability of 10% formalin or 4% PFA to inactivate samples. If inactivation was incomplete due to high cell numbers or short incubation times, this was generally observed for both formalin and PFA treatment. Adhering to longer incubation times for aldehyde inactivation is a standard method for tissue samples obtained from virus-infected animals. For filoviruses, it was shown that a 7-day incubation of 150 mg tissue from EBOV-infected animals in 10% formalin or 2% PFA at 4 °C led to complete virus inactivation [43]. Virus inactivation was also observed for ≤1 g of EBOV-infected tissue incubated in 10% formalin for 2 and 7 days [44]. For NiV, it was reported that incubation of two half-infected mouse lungs in 10% formalin for 48 h with a formalin change after 24 h led to complete virus inactivation [36]. Similarly, kidney tissue from NiV-infected ferrets was inactivated after a 48-h incubation in 10% formalin [51]. With one exception, liver samples tested in the same study were completely inactivated after a 24-h incubation in 10% formalin. Of note, it has been reported that formaldehyde penetrates tissue quickly but fixes slowly, adding to the complexity of formaldehyde fixation [52]. In summary, the complete aldehyde inactivation of virus-containing tissue samples and cell pellets relies on numerous parameters, including physicochemical properties of the virus, viral load, sample type, sample volume, time for penetration and fixation, temperature, aldehyde concentration, etc. These parameters must be clearly defined for different sample types, and testing for each condition must be performed to validate inactivation.

To ensure the sensitivity of the methods used to detect virus, each of our inactivation studies was accompanied by a limit of detection analysis. We detected 100 TCID_50_ units of infectious HAZV-GFP across all replicates from all experiments. This is in line with our previous inactivation studies, where we showed the reliable detection of a range of viruses, including EBOV, NiV, LASV, and SARS-CoV-2, using 10 or 100 TCID_50_ units for infection [33,34,35]. In three out of six experiments, we were able to detect 10 TCID_50_ units of HAZV-GFP, and in two out six experiments, we detected 1 TCID_50_ unit. The reason for this variation is based on statistical probability. Limit of detection studies are based on serial dilutions of a virus stock. The number of infectious viral particles in the highest dilutions is determined by statistical probability. At high dilutions, there could be 1, 5, or 50 infectious viral particles in a given dilution, or no infectious viral particles at all. In addition, the infection process itself is stochastic. Not every infectious viral particle in a solution will initiate a productive infection. This was determined by empirical and statistical analyses decades ago, leading to the application of Poisson distribution to viral infections [53]. These early virological analyses revealed that an MOI of 0.7 infectious particles per cell is required to infect 50% of the cells in a fully permissive cell monolayer and an MOI of 3 to infect 95% of the cells, which indicates that not every single infectious viral particle is able to start a productive infection [54]. For example, the viral particle might not be able to attach to a cell because the necessary attachment factors are not available on this specific cell. Or the infected cells could die before a productive viral infection was established, and there will be no progeny viruses. There are many reasons why a specific infection will not occur, which makes viral infection a statistical event. Therefore, working at the limit of detection (100, 10, or 1 TCID50 units) will inherently lead to some variability in the infection and viral detection process, as also demonstrated in this study.

Using BSL-2 surrogates for highly pathogenic viruses allows to streamline the validation of inactivation procedures and enables diagnostic laboratories without access to high and maximum containment laboratories to test inactivation methods for potentially infected incoming samples. This is particularly important for CCHFV because of its wide geographic distribution. BSL-2 surrogate viruses used for inactivation studies must meet specific standards. First, the surrogate virus must be closely related to the virus of interest and share physicochemical features that determine inactivation procedures. These include genome structure, viral particle complexity, and whether the virus is enveloped. For Select Agents, the surrogate virus and the virus of interest must be within the same virus family. Second, reliable methods must be available to detect infectious virus. In our case, we used a recombinant virus expressing a fluorescence protein, and we had access to an efficient virus-specific antibody. The induction of virus-induced CPE is desirable as it allows to easily distinguish between infected and uninfected cells. However, we only observed moderate CPE induced by HAZV-GFP infection in the Vero E6 cell line used. Third, high infection rates and high viral titers are desirable for performing inactivation studies as it allows for the inclusion of high safety margins. The titers of the HAZV-GFP virus stocks used in this study were in the range of 9 × 10^7^ TCID_50_ units per mL, which is more than 1 log higher than the titers we obtained for our CCHFV stocks, which were in the 10^6^ range. If necessary, virus stocks can be concentrated by, e.g., sucrose cushion ultracentrifugation or ultrafiltration [41,55]. Using BSL-2 viruses as surrogates for highly pathogenic viruses to test inactivation procedures is not a novel concept. For example, a comprehensive inactivation study utilized Morogoro virus, a nonpathogenic arenavirus, as a BSL-2 surrogate for LASV and other BSL-4 arenaviruses [41]. Likewise, vaccinia virus has been used as a surrogate for variola and mpox viruses to validate inactivation procedures for poxviruses [56,57,58]. Another concept is to use attenuated vaccine strains of the virus of interest for inactivation studies [42].

A few examples of suitable BSL-2 surrogates for select BSL-4 viruses are shown in Table 2. These viruses were selected based on their close relationship to the respective BSL-4 pathogens, suitable viral detection methods, and achievable high infection rates or viral titers. While there are a number of nonpathogenic arenaviruses that could be used as surrogates for inactivation studies [41,59,60,61,62], Cedar virus is currently the only suitable BSL-2 surrogate for BSL-4 henipaviruses [63,64] (Table 2). In contrast to henipaviruses, arenaviruses, and nairoviruses, there are currently no BSL-2 surrogates available for filoviruses. However, recent work on Bombali virus (BOMV) and Lloviu virus (LLOV), both members of the filovirus family, suggests that these viruses might be apathogenic for humans [39,65,66,67]. Recombinant systems that allow the introduction of fluorescent reporter genes into the viral genomes exist for both viruses [39,66]. Although more careful investigations are required to confirm the lack of pathogenicity for BOMV and LLOV, they are promising candidates for down classification to BSL-2 to serve as filovirus surrogates in inactivation studies. In conclusion, using BSL-2 viruses as surrogates for highly pathogenic BSL-4 viruses and Select Agents for inactivation is a viable approach as long as the surrogate viruses meet certain well-defined criteria.

## Figures and Tables

**Table 1:** Results of the limit of detection analysis. Positive replicates were determined by CPE, GFP-fluorescence, and virus-specific immunofluorescence analysis.

Virus	TCID_50_ Units	Number of Positive Replicates/ Total Number of Replicates
HAZV-GFP	1	2/6
	10	3/6
	100	6/6

**Table 2:** Examples of potential surrogates for inactivation studies of select BSL-4 viruses. NNS RNA, non-segmented negative-sense RNA; SNS, segmented negative-sense RNA. Number of genome segments is indicated. Note that some of the recombinant arenaviruses have 3 instead of 2 genome segments, which could affect their use in inactivation studies.

BSL-4 Viruses	Genome Organization	BSL-2 Surrogate	Expression of Fluorescent Protein	References
henipaviruses	NNS RNA	Cedar virus	yes	[63,64]
filoviruses	NNS RNA	no	N/A	
arenaviruses	SNS RNA 2 segments	Morogoro virus	no	[41]
		Mopeia virus	yes ^1^	[59]
		Tacaribe virus	yes	[60]
		Pichinde virus	yes ^1^	[61,62]
nairoviruses	SNS RNA 3 segments	HAZV	yes	[22,27]

^1^ Tri-segmented.

## Data Availability

Data are contained within the article or Supplementary Material.

## Supplementary Materials

The following supporting information can be downloaded at: https://www.mdpi.com/article/10.3390/pathogens14070700/s1. Figure S1: Initial infection rate for TRIzol inactivation testing; Figure S2: Inactivation of HAZV-GFP-infected cell monolayers with 4% PFA; Figure S3: Inactivation of HAZV-GFP-infected cell pellets with 4% PFA.
