# Supplementary material for: Harnessing Hazara Virus as a Surrogate for Crimean–Congo Hemorrhagic Fever Virus Enables Inactivation Studies at a Low Biosafety Level
Source: Pathogens. 2025 Jul 15;14(7):700. doi: 10.3390/pathogens14070700 (PMC12300750; doi:10.3390/pathogens14070700)
Supplement: Supplementary file 1 [file pathogens-14-00700-s001.zip › pathogens-3611664-supplementary.pdf]

Harnessing Hazara virus as a surrogate for Crimean Congo hemorrhagic fever virus enables inactivation studies at low biosafety level

Judith Olejnik [1,2], Kristina Meier [1,2], Jarod N. Herrera [1,2,], Daniel J. DeStasio [1,2], Dylan J. Deeney [1,2], Elizabeth Y. Flores [2, 3], Mitchell R. White [1,2], Adam J. Hume [1,2,*], and Elke Mühlberger [1,2,*]

[1] Department of Virology, Immunology and Microbiology, Chobanian & Avedisian School of Medicine, Boston University, Boston, MA 02118
[2] National Emerging Infectious Diseases Laboratories (NEIDL), Boston University, Boston, MA 02218
[3] Center for Regenerative Medicine (CReM), Boston University and Boston Medical Center, Boston, MA 02118, USA

* Correspondence: hume@bu.edu, Tel.: +1 617-358-9151; muehlber@bu.edu, Tel.: +1 617-358-9153

Supplementary Figures

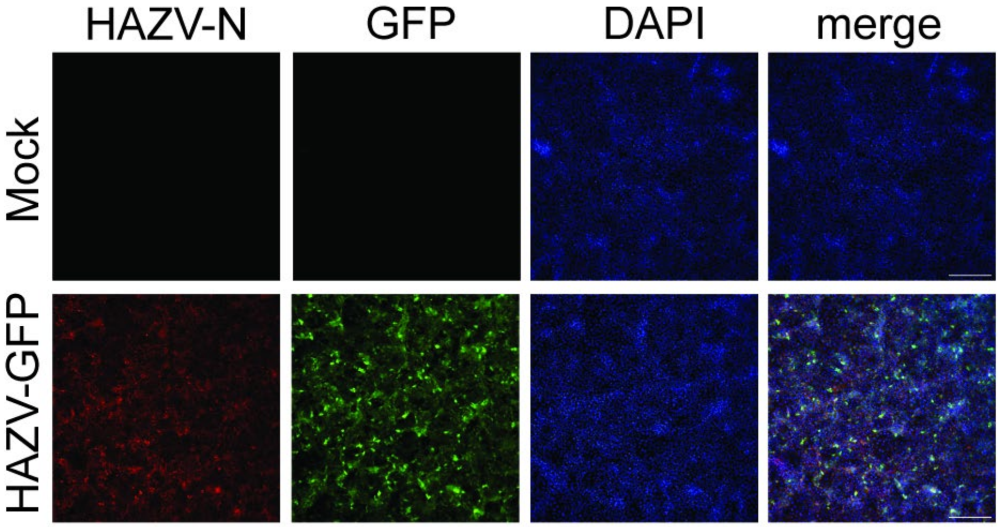

Figure S1. Initial infection rate for TRIzol inactivation testing. Slides were mock infected or infected with HAZV-GFP at an MOI of 0.05. At 3 dpi slides were fixed at time of inactivation of test samples. Fluorescence microscopy and IFA were performed. Infected cells show clear GFP-expression (green) and staining for HAZV-N (red). Green, GFP expressing cells; red, virus-specific antibody (HAZV-N) staining; blue, cell nuclei stained with DAPI. Scale bars, 250 μm. The initial infection rates for the aldehyde inactivation studies were determined by the same approach, also resulting in near complete infection. Experiment was performed twice with similar outcome for the TRIzol inactivation study. Initial infection rates were determined for each inactivation study resulting in 6 experiments with similar outcome.

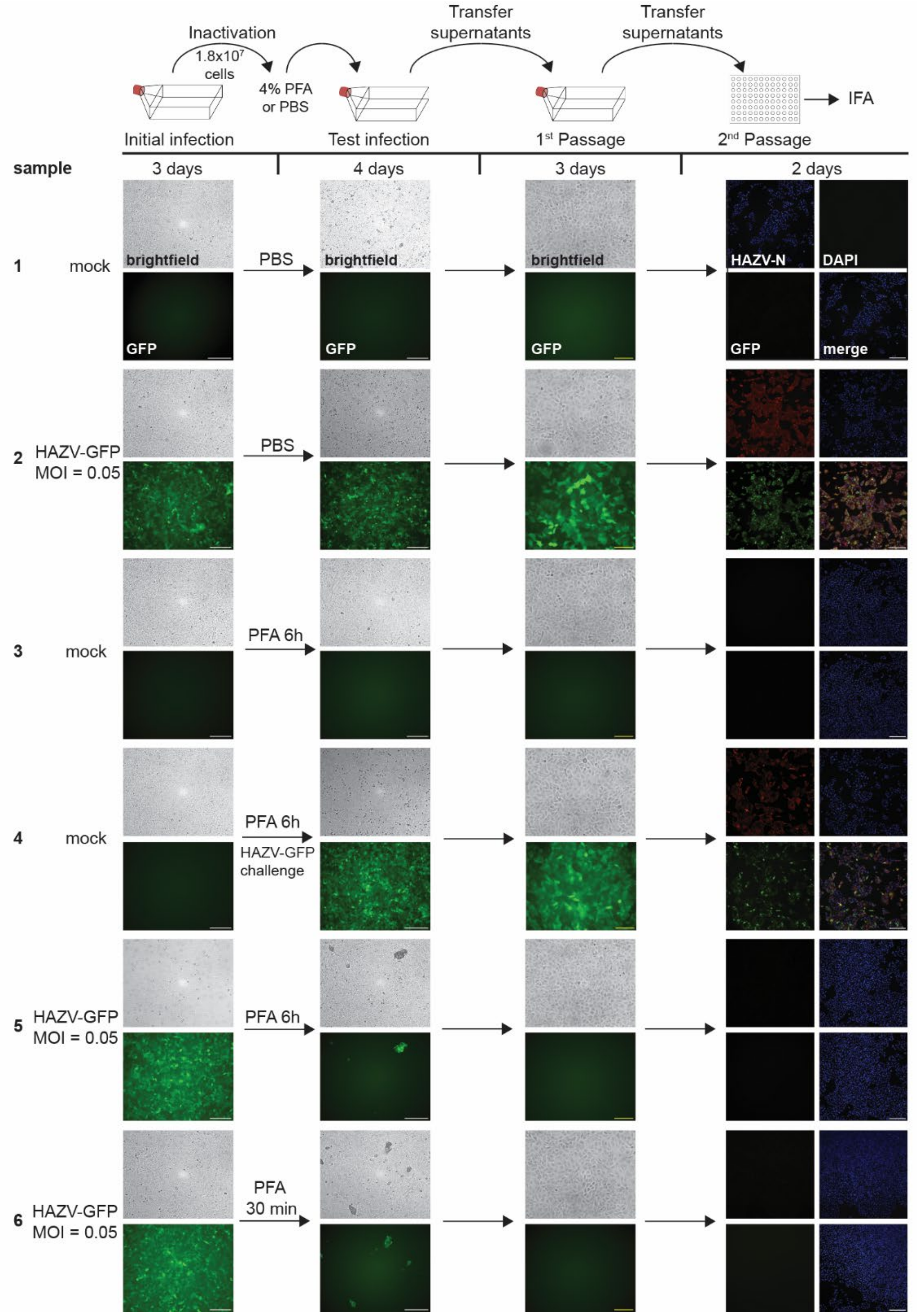

Figure S2. Inactivation of HAZV-GFP-infected cell monolayers with 4% PFA. Top, schematic of the assay. Vero E6 cells seeded in T175 flasks were mock infected or infected with HAZV-GFP at an MOI of 0.05. At 3 dpi, fluorescence and brightfield images were taken to assess GFP fluorescence and CPE as markers for viral infection (Initial infection). Cells were incubated with 4% PFA or PBS (control). Treated cells were washed, pelleted, and transferred onto Vero E6 cells seeded in T175 flasks (Test infection). Challenge samples were infected with HAZV-GFP at an MOI of 0.05. At 4 dpi, clarified supernatants were passaged onto Vero E6 cells seeded in T175 flasks. Cells were incubated for additional 3 days and monitored for viral infection (1st Passage). Note that images for 1st passage were taken at higher magnification. Clarified

supernatants were then used to infect Vero E6 cells seeded in 96-well plates and fixed at 2 dpi. IFA was performed using an anti-HAZV N antibody (red, 2nd Passage). Cell nuclei were stained with DAPI (blue). White scale bars, 250 µm; yellow scale bars in 1st passage column, 100 µm. Image labeling for all samples as shown for sample 1. Images for samples 1 and 2 are also shown if Figure 5 because formalin and PFA fixation were done in parallel using the same mock and infection controls. The experiment was performed twice with similar outcome.

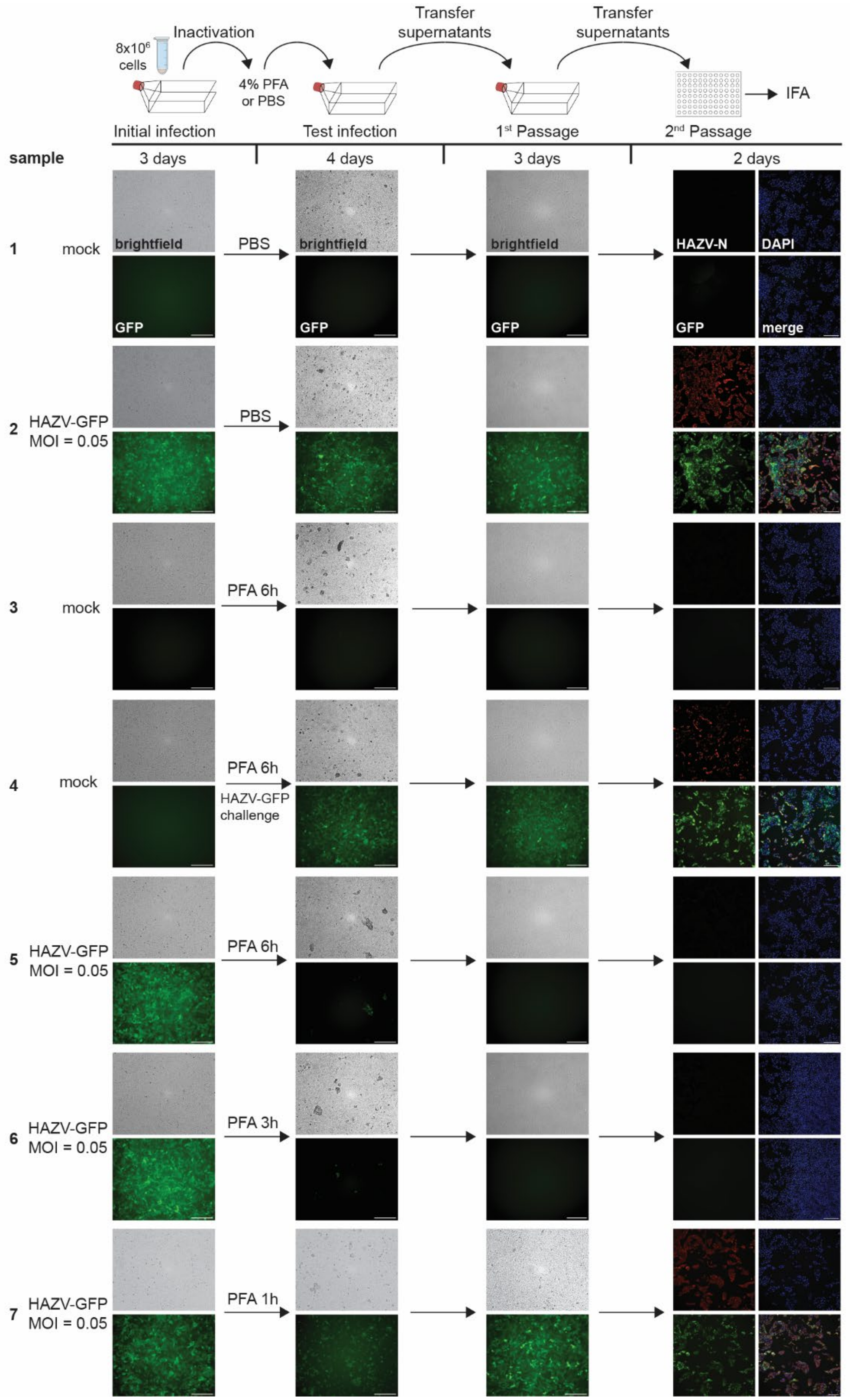

Figure S3. Inactivation of HAZV-GFP-infected cell pellets with 4% PFA. Top, schematic of the assay. Vero E6 cells seeded in T175 flasks were mock infected or infected with HAZV-GFP at an MOI of 0.05. At 3 dpi, fluorescence and brightfield images were taken to assess the presence

of GFP and CPE in samples as a marker for viral infection (Initial infection). Cells were pelleted and overlayed with 4% PFA or PBS (control) for fixation. After removal of the fixative, cell pellets were washed and transferred onto Vero E6 cells seeded in T175 flasks (Test infection). Challenge samples were infected with HAZV-GFP at MOI 0.05. At 4 dpi, clarified supernatants were passaged onto Vero E6 cells seeded in T175 flasks (1st Passage). At 3 dpi, clarified supernatants were then used to infect Vero E6 cells seeded in 96-well plates and fixed at 2 dpi. IFA was performed using an anti-HAZV N antibody (red, 2nd Passage). Cell nuclei were stained with DAPI (blue). Scale bars, 250 μm. Image labeling for all samples as shown for sample 1. Images for samples 1 and 2 are also shown if Figure 6 because formalin and PFA fixation were done in parallel using the same mock and infection controls. The experiment was performed twice with similar outcome.
